# Relationship between osteoporosis and Cushing syndrome based on bioinformatics

**DOI:** 10.1097/MD.0000000000031283

**Published:** 2022-10-28

**Authors:** Ding Wang, Chun-Xiao Dang, Ying-Xin Hao, Xiao Yu, Peng-Fei Liu, Jin-Song Li

**Affiliations:** a Shandong University of Traditional Chinese Medicine, Jinan, China; b Affiliated Hospital of Shandong University of Traditional Chinese Medicine, Jinan, China; c Anqiu Hospital of Traditional Chinese Medicine, Weifang, China.

**Keywords:** bioinformatics, Cushing syndrome, interrelationships, osteoporosis

## Abstract

Many clinical studies have reported a relatively high incidence of osteoporosis and fragility fractures in patients with Cushing syndrome (CS). However, few papers have investigated osteoporosis and CS in terms of pathogenesis, so this study explores the association between the 2 and predicts upstream micro-ribonucleic acids (miRNAs) through bioinformatics, which provides potential targets for simultaneous pharmacological interventions in both diseases and also provides a basis for pathological screening.

**Methods:**

We used Genecards, Online Mendelian Inheritance in Man and Therapeutic Target Database databases to screen the targets of osteoporosis and Cushing syndrome; import target genes to Database for Annotation, Visualization and Integrated Discovery for Gene Ontology and Kyoto Encyclopedia of Genes and Genomes pathway analysis; the intersecting genes were uploaded to Search Tool for the Retrieval of Genes and Genomes database to construct protein–protein interaction network; Cytoscape software was used to screen core genes, and Molecular Complex Detection module was used to analyze cluster modules; finally, the NetworkAnalyst data platform was used to predict the miRNAs that interact with core genes.

**Results:**

The core genes of osteoporosis and Cushing syndrome were insulin, tumor necrosis factor, signal transducer and activator of transcription 3 (STAT3), interleukin-6, insulin-like growth factor 1, etc. A total of 340 upstream miRNAs including hsa-let-7a-5p, hsa-mir-30a-5p and hsa-mir-125b-5p were predicted. The biological processes involved include regulating the transcription of ribonucleic acid polymerase II promoter and participating in the transduction of cytokine signaling pathways, which focus on the binding of nerve system ligand, JAK-STAT signaling pathway, Rap1 signaling pathway, PI3K-Akt signaling pathway, etc.

**Conclusion:**

Osteoporosis and Cushing syndrome are closely related in terms of targets and molecular mechanisms. In this study, bioinformatics methods were used to identify their targets and mechanisms, providing potential targets for drug simultaneous regulation of the 2 diseases, and providing a new direction for exploring the relationship between diseases.

## 1. Introduction

Osteoporosis is a systemic skeletal disease characterized by a decrease in bone density and bone mass, and the associated factors are complex and diverse.^[1]^ Cushing syndrome is a group of symptoms and signs caused by abnormally elevated cortisol from multiple causes, and can be divided into Cushing disease of pituitary origin (e.g., pituitary adenoma) and Cushing disease of adrenal origin (e.g., adrenocortical adenoma, adrenal nodular hyperplasia, etc.), which is an important cause of secondary osteoporosis.^[2]^ The prevalence of bone loss and osteoporosis in Cushing syndrome (CS) patients has been reported to be 60% to 80%, 30% to 65%, respectively.^[3,4]^ The incidence of fragility fracture is 30% to 50%.^[4]^

Therefore, it is urgent to clarify the relationship between osteoporosis and Cushing syndrome and to reduce the risk of osteoporosis in CS patients. Integration of genetic data from osteoporosis and Cushing syndrome using bioinformatics to explore the association and provide targets for simultaneous pharmacological interventions in both diseases, as well as a theoretical basis for pathological screening.

## 2. Materials and Methods

### 2.1. Collection of relevant target genes for osteoporosis-Cushing syndrome

The keywords “Osteoporosis” and “Cushing syndrome” were used to search for Genecards (https://www.genecards.org/),^[5]^ Online Mendelian Inheritance in Man (http://www.omim.org),^[6]^ Therapeutic Target Database (http://db.idrblab.net/ttd/)^[7]^ databases to screen for disease-related targets. To obtain high accuracy, the top 300 genes with high correlation scores were selected from the Genecards database. The results were combined and de-duplicated and then screened through the Uniprot database to identify the clearly certified target genes among them as the final target genes for osteoporosis and Cushing syndrome, and finally mapped the target genes for osteoporosis and Cushing syndrome to obtain the intersecting genes.

### 2.2. Gene function enrichment analysis

The target genes of osteoporosis and Cushing syndrome were imported into the Database for Annotation, Visualization and Integrated Discovery (https://david.ncifcrf.gov/) database, respectively.^[8]^ And set *P* < .05, FDR < 0.05 for Gene Ontology (GO), Kyoto Encyclopedia of Genes and Genomes (KEGG) enrichment analysis.^[9]^ The top 3 GO pathways and the top 5 KEGG pathways were screened by *P* value from smallest to largest, respectively.

### 2.3. PPI network and module analysis

Search Tool for the Retrieval of Genes and Genomes (STRING) (https://string-db.org/cgi/input.pl) database can be used to present and evaluate protein interaction relationships.^[10]^ The intersecting genes of osteoporosis and Cushing syndrome were uploaded to STRING database to construct protein–protein interaction (PPI) network, download the tsv format of PPI network, import it into Cytoscape 3.8.0 software, (https://cytoscape.org/), and use network analyzer plug-in to perform node connectivity (degree), combined score (combined score) based on visual representation. Finally, the top 10 genes were selected as the core target genes for osteoporosis-Cushing syndrome, and the parameters were set as degree = 2, node score = 0.2, k-core = 2, and max. depth = 100 for cluster module analysis of the intersecting genes.^[11]^

### 2.4. Prediction of micro-ribonucleic acid (miRNA)-gene correspondence

NetworkAnalyst 3.0 (https://www.networkanalyst.ca/) is a platform that allows online gene expression analysis for differential gene screening, functional enrichment analysis, and gene-miRNA network construction.^[12]^ In this study, the NetworkAnalyst 3.0 platform, miRTarBase database was used to predict miRNAs that may interact with core target genes.

## 3. Results

### 3.1. Results of target genes collection for osteoporosis-Cushing syndrome

**Figure 1.**
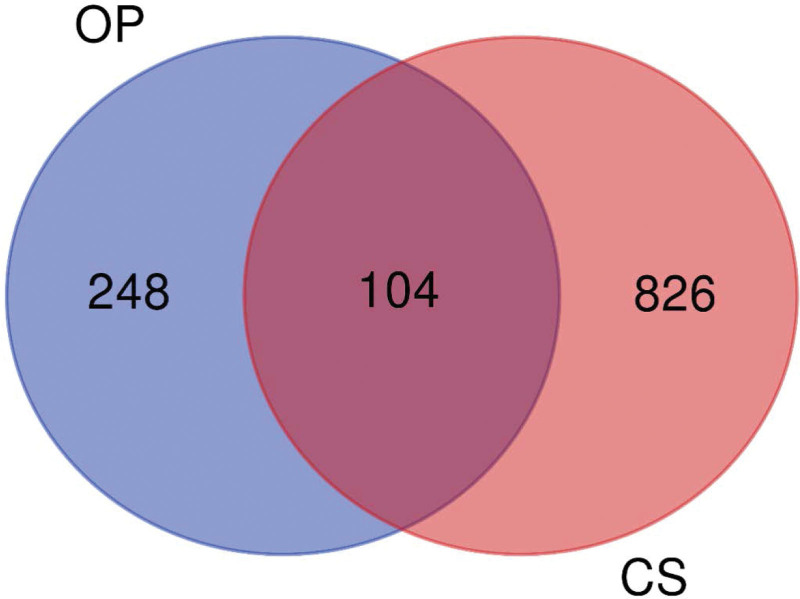
Intersection target of OP and CS. CS = Cushing syndrome, OP = osteoporosis.

The disease target genes of osteoporosis and Cushing syndrome were collected from Genecards, Online Mendelian Inheritance in Man and Therapeutic Target Database databases respectively, and the validated target genes were screened by Uniprot database, and finally the results of the 3 databases were combined and de-duplicated to obtain a total of 352 validated target genes for osteoporosis and 930 validated target genes for Cushing syndrome, with a total of 104 intersecting genes between the 2 diseases (Fig. 1).

### 3.2. Results of GO items and KEGG analysis

**Figure 2.**
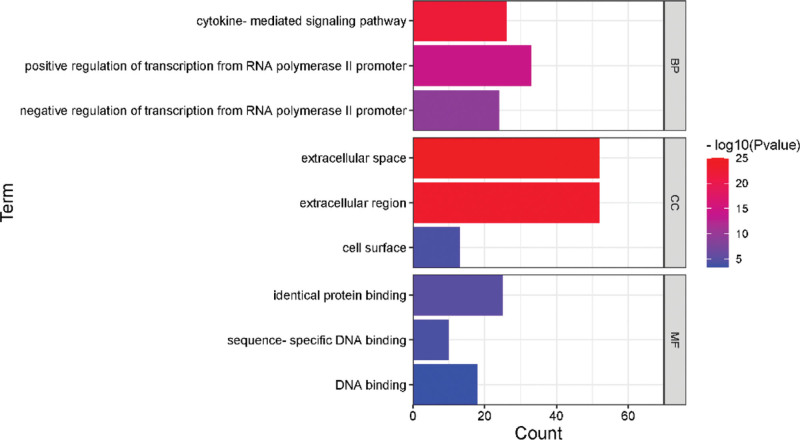
The first 3 GO pathways of OP and CS. CS = Cushing syndrome, GO = gene ontology, OP = osteoporosis.

**Figure 3.**
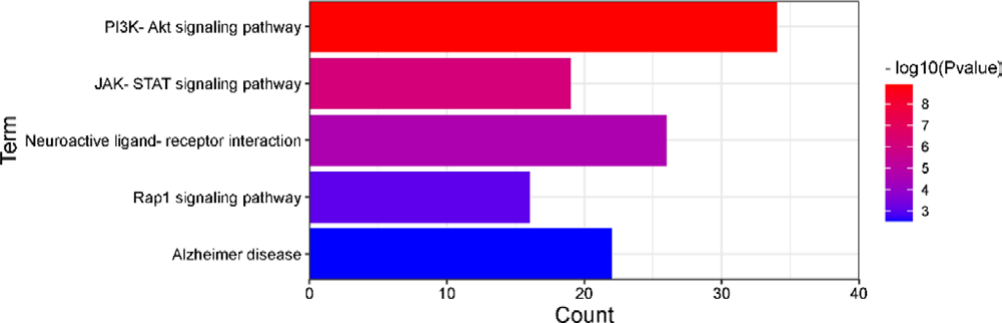
The first 5 KEGG pathways of OP and CS. CS = Cushing syndrome, KEGG = Kyoto Encyclopedia of Genes and Genomes, OP = osteoporosis.

GO analysis can classify and annotate genes according to biological process, molecular function and cellular component and identify their biological properties, while KEGG pathway analysis is a common method for gene function enrichment analysis. The GO and KEGG pathway analysis of osteoporosis and Cushing syndrome was performed by the Database for Annotation, Visualization and Integrated Discovery database, and a total of 136 and 168 KEGG pathways were obtained for osteoporosis and Cushing syndrome, and a total of 280 and 557 GO entries were obtained, including 241 and 434 for biological process, 7 and 54 for cellular component, and 32 and 69 for molecular function, respectively. The top 3 GO entries with relatively small *P* value among those meeting both *P* < .05 and FDR < 0.05 were screened as Figure 2. The results showed that high relevance in biological processes such as regulation of transcription of ribonucleic acid polymerase II promoter and participation in transduction of cytokine signaling pathway. At the cellular level, mainly enriched in the cell surface and extracellular regions. At the molecular level, it had the function of promoting DNA and protein binding. Using *P* < .05 as the screening condition, the results of KEGG analysis were arranged in ascending order of *P* value, and the top 5 enriched pathways common to both diseases were selected as Figure 3. The results showed that the genes were mainly enriched in the binding of neurological receptor ligands, JAK-STAT signaling pathway, Rap1 signaling pathway and PI3K-Akt signaling pathway.

### 3.3. Core gene screening and cluster module analysis

**Figure 4.**
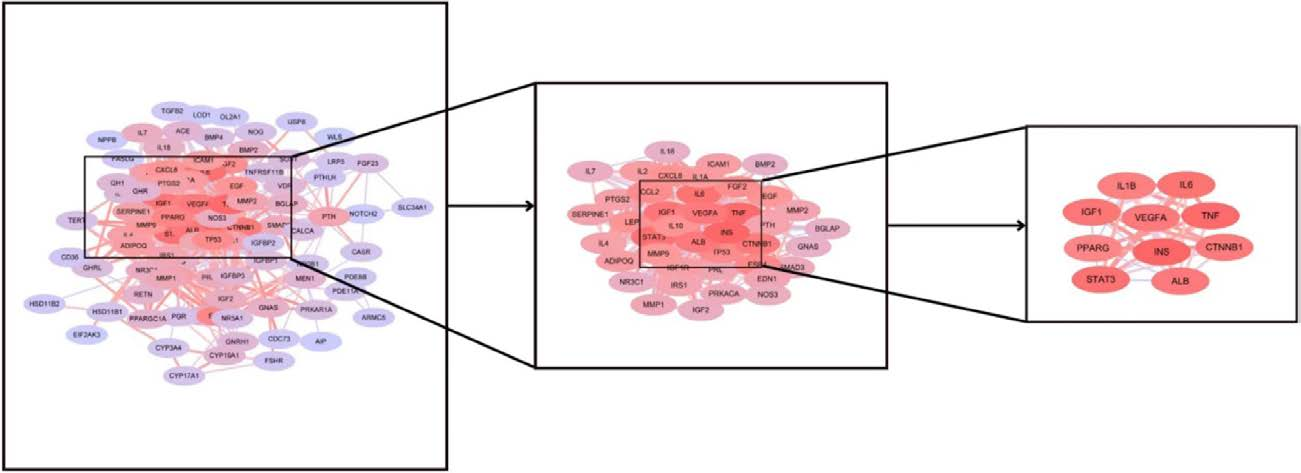
Top ten core target genes of OP and CS. CS = Cushing syndrome, OP = osteoporosis.

**Figure 5.**
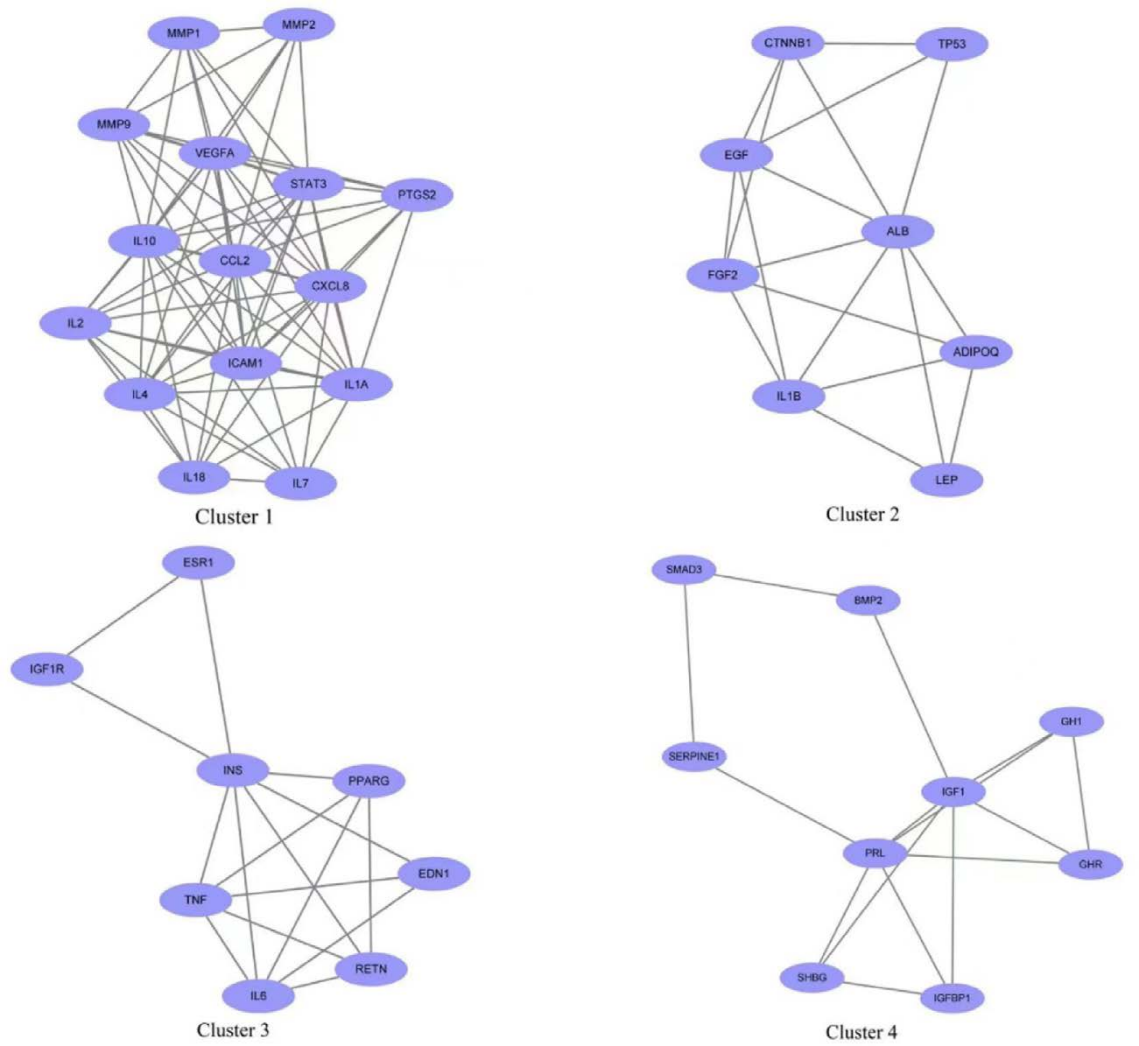
Cluster module analysis diagram.

The intersection genes of osteoporosis and Cushing syndrome were uploaded to STRING database, the minimum interaction score was set to 0.7 and the free nodes were hidden to generate PPI network. The results showed that there were 102 target genes with interconnection, the network had 561 edges, the average node degree was 11 and the average clustering coefficient was 0.511. The higher the node connection degree, the more the gene was connected to other genes, and the higher the possibility of becoming a core gene, so its tsv file was downloaded and imported into Cytoscape 3.8.0 software, and the top 10 genes were selected as the core target genes for both diseases according to node connectivity (Degree) from highest to lowest: INS (degree: 38), tumor necrosis factor (TNF) (degree: 36), signal transducer and activator of transcription 3 [STAT3 (degree: 34)], interleukin-6 (IL6) (degree: 33), insulin-like growth factor 1 [IGF1 (degree: 32)], ALB (degree: 31), VEGFA (degree: 31), CTNNB1 (degree: 30), IL1B (degree: 28), PPARG (degree: 26) (Fig. 4). The cluster module analysis can explore the most closely linked gene clusters in the PPI network. We selected the top 4 clusters with the highest score and analyzed the enriched pathways of each cluster: Cluster1 and Cluster4 were mainly enriched in the interaction between cytokines and their receptors; Cluster2 was mainly enriched in the MAPK signaling pathway; Cluster3 was mainly enriched in the HIF-1 signaling pathway (Fig. 5).

### 3.4. Identification of miRNA–gene relationships

**Figure 6.**
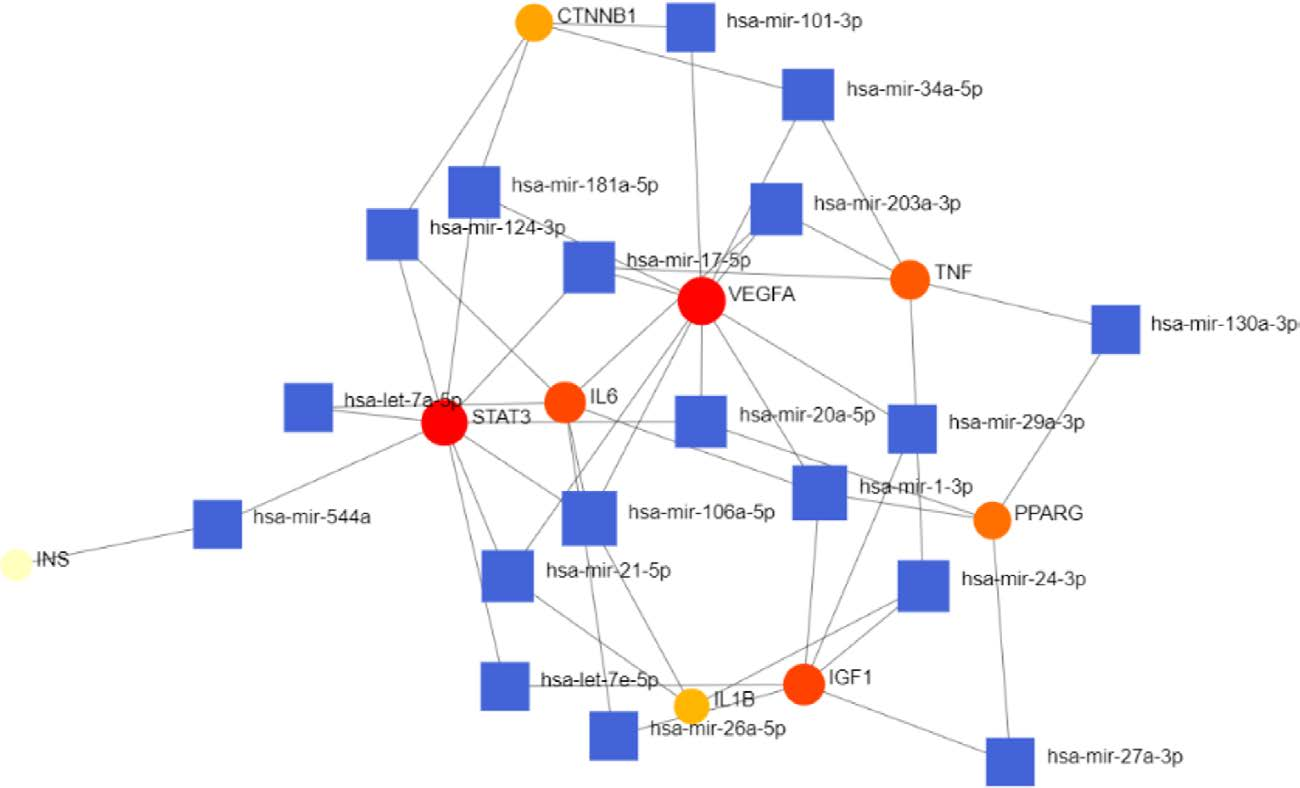
miRNA-core gene analysis figure. miRNA = micro-ribonucleic acid.

In this study, the NetworkAnalyst 3.0 platform was applied to predict miRNAs that may interact with the above 10 core genes. The results showed that a total of 340 miRNAs interacted with 9 core genes. For high connectivity, 18 miRNAs with interactions were further screened, as shown in Figure 6. The top 10 miRNAs and their corresponding relationships were ranked according to the number of genes corresponding to miRNAs in descending order, as shown in Table 1.

**Table 1:** miRNA-core gene mapping table.

miRNA	Gene
hsa-mir-106a-5p	STAT3 IL6 VEGFA IL1B
hsa-mir-1-3p	IL6 VEGFA PPARG IGF1
hsa-mir-17-5p	STAT3 VEGFA TNF
hsa-mir-20a-5p	STAT3 VEGFA PPARG
hsa-mir-21-5p	STAT3 VEGFA IL1B
hsa-mir-24-3p	TNF IL1B IGF1
hsa-mir-34a-5p	VEGFA CTNNB1 TNF
hsa-mir-181a-5p	STAT3 VEGFA CTNNB1
hsa-mir-203a-3p	IL6 VEGFA TNF
hsa-mir-124-3p	STAT3 IL6 CTNNB1

IL6 = interleukin-6, miRNA = micro-ribonucleic acid, TNF = tumor necrosis factor.

## 4. Discussion

### 4.1. Results and analysis

Clinical studies^[13,14]^ have found that osteoporosis and Cushing syndrome are 2 closely related diseases, and many CS patients have decreased serum osteocalcin levels,^[15]^ even with multiple fragility fractures as the first diagnosis.^[16]^ Therefore, this study used bioinformatics to search for cross sections and common pathways in the vast gene networks of these 2 diseases, and predicted 340 upstream miRNAs, including 10 more critical miRNAs such as hsa-let-7a-5p, hsa-mir-30a-5p, and hsa-mir-125b-5p, which could provide potential targets for drug therapy. A total of 104 intersecting target genes were found between osteoporosis and Cushing syndrome, accounting for about 8.83%, with core target genes such as INS, TNF, STAT3, IL6, IGF1, etc.; the biological processes involved include regulation of ribonucleic acid polymerase II promoter transcription, involvement in cytokine signaling pathway conduction, etc.; KEGG pathway analysis showed that the 2 diseases involved neurological ligand recipient interaction pathway, JAK-STAT signaling pathway, Rap1 signaling pathway, PI3K-Akt signaling pathway and many other signaling pathways.

Cushing syndrome is often associated with insulin (INS) resistance, and its complication rate of diabetes is as high as 43.3%.^[17]^ INS is a peptide hormone secreted by pancreatic beta cells, which can maintain the stability of blood glucose level and reduce the apoptosis rate of osteoblasts through various glucose metabolism pathways such as type I diabetic signaling pathway.^[18]^ It can also bind to INS receptors on the surface of osteoblasts and promote the proliferation and differentiation of osteoblasts.^[19]^ TNF and IL6, as pro-inflammatory cytokines, play important roles in regulating lipid metabolism and INS signaling, and can also mediate bone resorption.^[20]^ TNF-α, a major pro-inflammatory factor, is highly expressed in the subcutaneous adipose tissue of CS patients, and its synergistic inflammatory response with IL6 can promote osteoclast maturation and differentiation and inhibit osteoprotegerin synthesis,^[21,22]^ leading to osteoporosis. STAT3 promotes the proliferation and differentiation of Th17 cells, the secretion of IL-17 and TNF-α,^[23]^ regulates osteoclast formation and bone resorption process, and inhibits osteoblast apoptosis by promoting the expression of the apoptosis suppressor protein Bcl-2.^[24]^ Research^[25,26]^ found that STAT3 is highly expressed in all types of pituitary adenomas, which can cause persistent high expression of FGF and vascular endothelial growth factor (VEGF) through the STAT3/PTTG signaling pathway, promoting the formation of tumor blood vessels and aggravating the condition of CS patients, while VEGF can help the proliferation and differentiation of osteoblasts, promote the recruitment and survival of osteoclasts, and promote bone formation. IGF1, as an osteogenic differentiation factor, can upregulate the expression of protein kinase and alkaline phosphatase mesenchymal stem cells, and participate in cartilage anabolism and repair.^[27]^ Animal experiments^[28]^ found that different concentrations of IGF1 played a positive role in regulating the expression of both cartilage layer and subchondral bone genes. In contrast, excessive INS promoted IGF1 overexpression, enhanced 17α-hydroxylase activity, and increased androgen synthesis and secretion in CS patients.^[29]^

The JAK-STAT signaling pathway is composed of the JAK family and STAT family, and many inflammatory cytokines such as IL-6 interact with their receptors to upregulate the differentiation of osteoblasts and osteoclasts through the JAK-STAT signaling pathway, affecting the production of bone trabeculae and regulating bone metabolism.^[30,31]^ Rap1 signaling pathway is mainly involved in the regulation of bone resorption, and its expression in mature osteoclasts can cause pathological bone loss.^[32]^ The PI3K-Akt signaling pathway, an important pathway regulating cell proliferation, differentiation and metabolism, promotes angiogenesis and proliferation of tumor cells in pituitary adenomas and inhibits apoptosis.^[33]^ It also affects bone metabolism by controlling the proliferation and differentiation of osteoblasts and osteoclasts, and is a key pathway affecting the coordination of osteoblast and osteoclast functions.^[34]^ Animal experiments^[35–37]^ confirmed that activation of the PI3K/AKT signaling pathway reduces estrogen and testosterone levels, thereby regulating osteoblast differentiation and apoptosis of osteoclasts. The neurological receptor-ligand interaction pathway, a collection of neuroactive receptors located on the plasma membrane, maintains the stability of the neuroendocrine system and has been shown to be associated with the development of pituitary adenomas in studies using microarray data.^[38,39]^

## 5. Conclusions

In this study, we used bioinformatics to search for common target genes and pathways between osteoporosis and Cushing syndrome, and found that INS resistance and the resulting high glucose levels in Cushing syndrome can affect the proliferation and differentiation of osteoblasts and apoptosis, while excessive INS causes overexpression of IGF1, which not only affects cartilage synthesis but also aggravates hormonal disorders. STAT3 and VEGF, on the other hand, induce angiogenesis in pituitary adenomas and also play a positive role in bone growth and reconstruction. TNF, IL-6 and IL-17-mediated inflammatory responses can inhibit osteoprotegerin synthesis and promote osteoclast differentiation, in addition to having an effect on glucose and lipid metabolism. The neurological ligand recipient interaction pathway, JAK-STAT signaling pathway, Rap1 signaling pathway, and PI3K-Akt signaling pathway play direct or indirect roles in this. And the prediction of core genes and upstream miRNAs not only provides potential targets for simultaneous drug intervention in both diseases, but also provides a basis for pathological screening and genetic diagnosis. The results of these analyses not only provide theoretical support for subsequent experimental studies, but the analysis methods embodied also serve to save resources and improve efficiency. Since this study is only a database based analysis, and the reliability and accuracy of the data will continue to improve with the continuous development of bioinformatics and disease databases, studies from cellular and animal testing are still needed to make the conclusions more reliable.

## Author contributions

**Conceptualization:** Ding Wang, Jin-Song Li.

**Data curation:** Chun-Xiao Dang, Ding Wang, Ying-Xin Hao.

**Formal analysis:** Chun-Xiao Dang, Ding Wang.

**Funding acquisition:** Peng-Fei Liu, Xiao Yu.

**Investigation:** Ding Wang, Peng-Fei Liu.

**Methodology:** Ding Wang, Xiao Yu, Ying-Xin Hao.

**Writing – original draft:** Ding Wang, Chun-Xiao Dang, Ying-Xin Hao.

**Writing – review & editing:** Peng-Fei Liu, Jin-Song Li.
